# Supplementary figures and images for: O-GlcNAc transferase plays a non-catalytic role in C. elegans male fertility
Source: PLoS Genet. 2022 Nov 16;18(11):e1010273. doi: 10.1371/journal.pgen.1010273 (PMC9710795; doi:10.1371/journal.pgen.1010273)

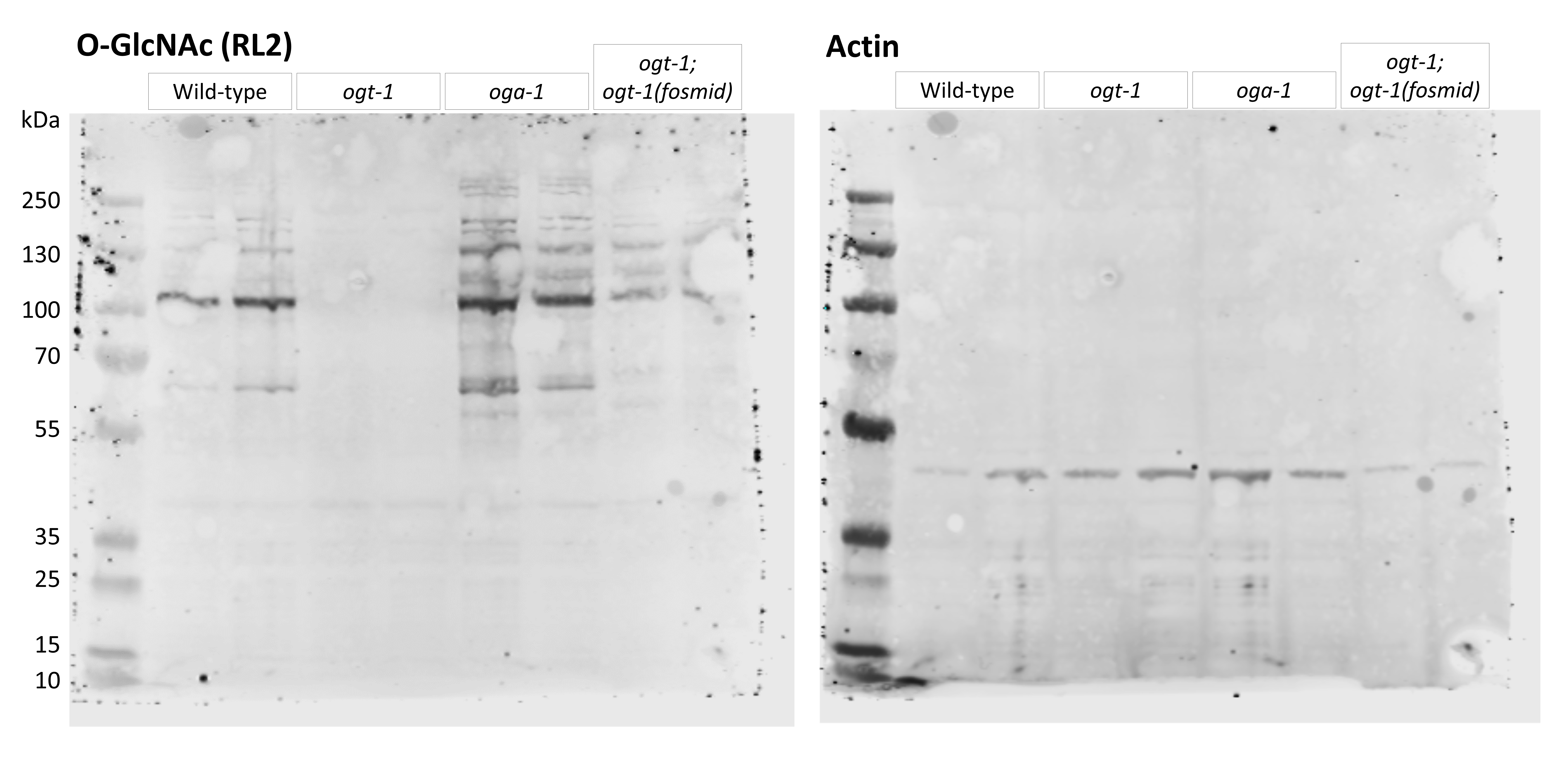

Supplement: S1 Fig — Left: O-GlcNAc (RL2 antibody), right: actin antibody. All genotypes have him-5 in background. (TIF) [file pgen.1010273.s001.TIF]

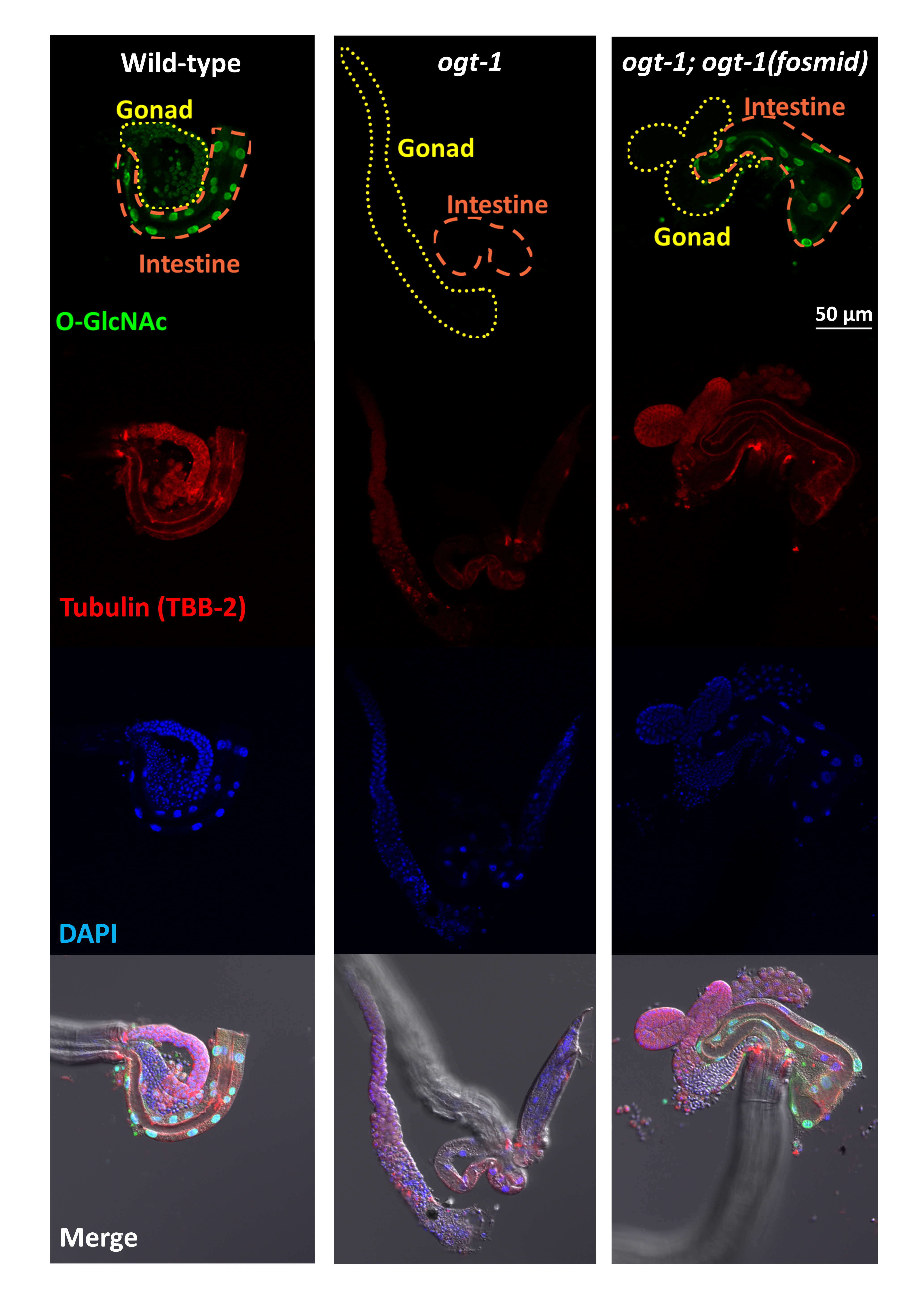

Supplement: S2 Fig — Representative immunohistochemistry images of dissected males, as shown in Fig 1D, including O-GlcNAc (RL2 antibody, in green), tubulin (anti-TBB-2 antibody, in red), DAPI (in blue), and all three fluorescent channels merged with differential interference contrast (DIC, greyscale). All worms are in the him-5 background. (TIF) [file pgen.1010273.s002.TIF]

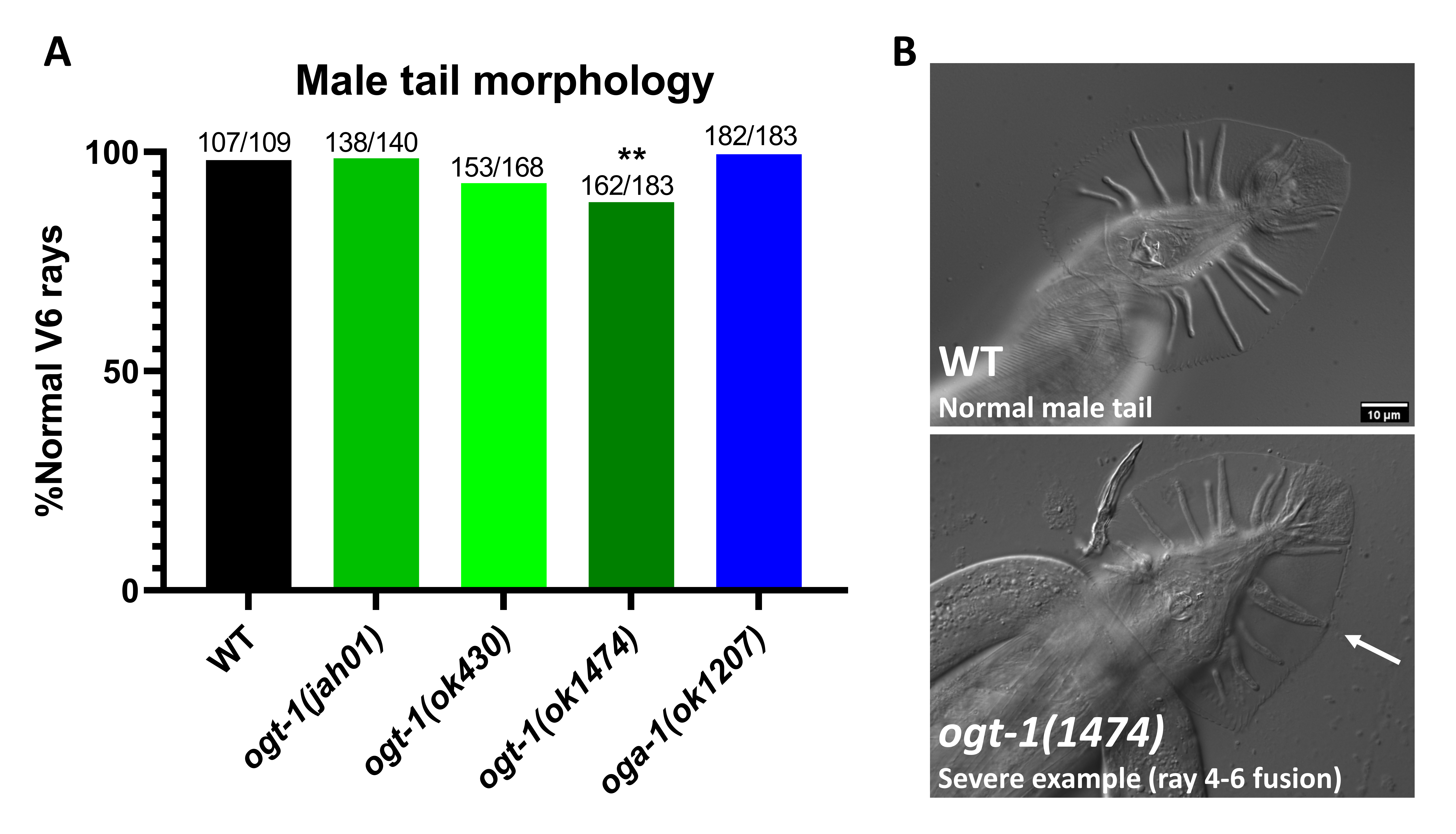

Supplement: S3 Fig — (A) Percentage of males with normal V6 ray morphology, with total number of observed normal and total males of each genotype. Defects include ray fusions and missing rays. Ratio of normal to deviant males was compared to wild-type with Fisher’s exact test and is shown above. ** = p<0.01, all others not significant. (B) Representative differential interference contrast (DIC) image of a normal male tail for wild-type control and a severe example of fused rays (arrow) in the ogt-1(ok1474) line. All males are in the him-5 background. (TIF) [file pgen.1010273.s003.TIF]

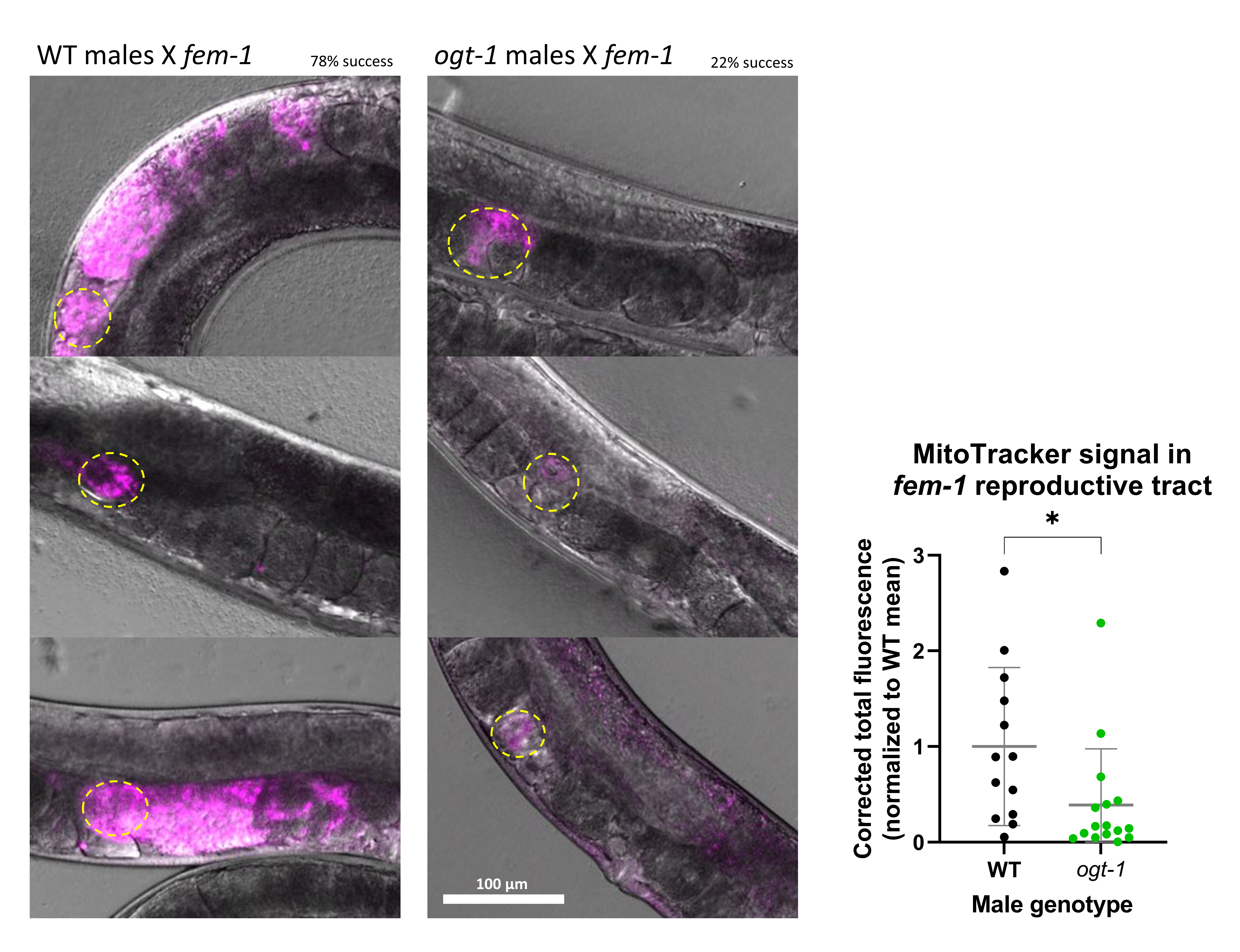

Supplement: S4 Fig — Three representative differential interference contrast DIC (greyscale) images of fem-1 animals after successful sperm transfer from MitoTracker-labelled (magenta) males of indicated genotypes. Yellow dashed lines indicate spermathecae. These images are examples of successful sperm transfer, excluding those with no sperm in their reproductive tract. Percent success rate (as shown in Fig 2C) is included with genotype above images. Right, quantification of MitoTracker signal in the reproductive tract of fem-1 worms after 1h mating, including only data from images of successful sperm transfer. (TIF) [file pgen.1010273.s004.TIF]

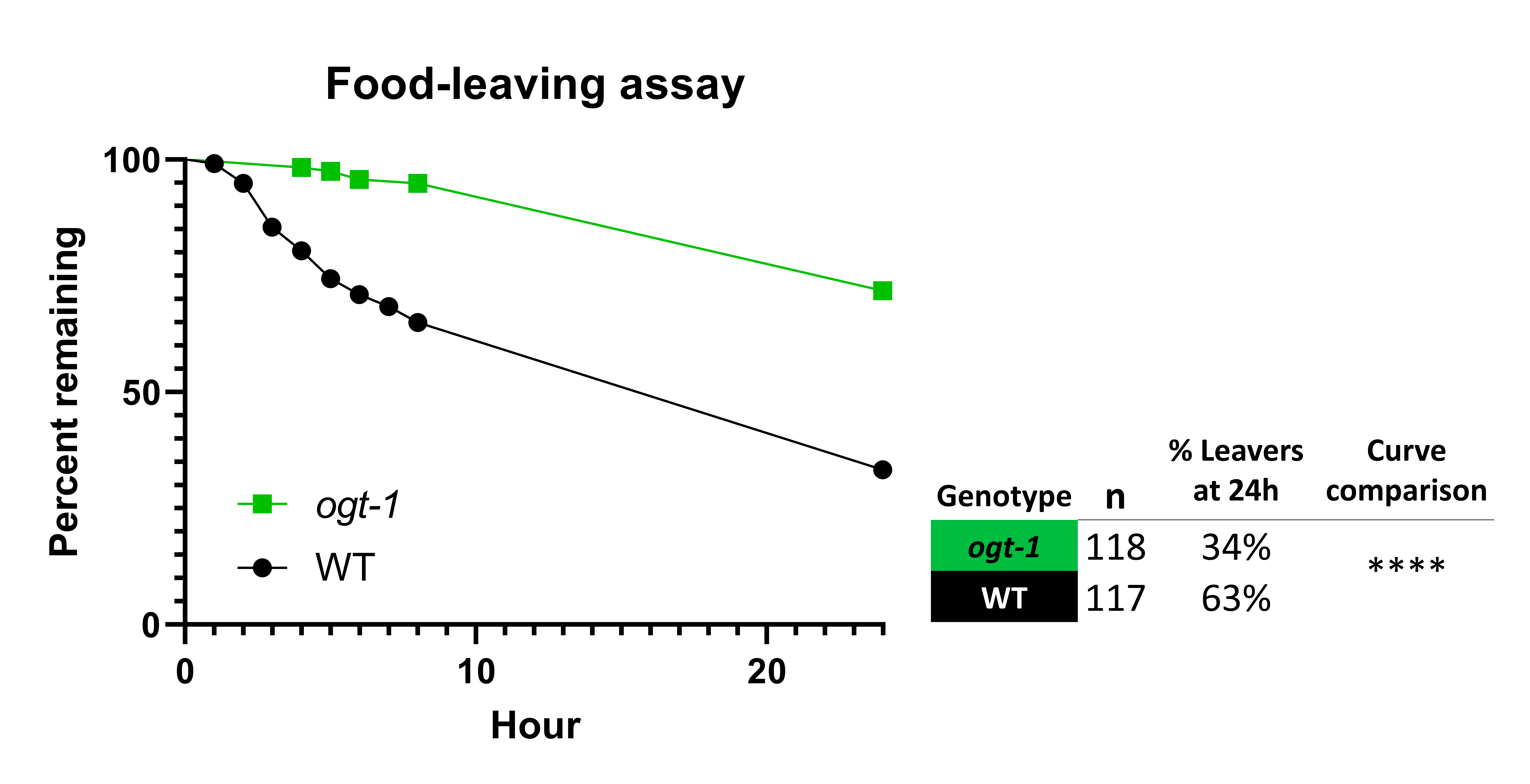

Supplement: S5 Fig — Time course of the food-leaving assay, of which the 24h timepoint is shown in Fig 3B. Each point represents the percent remaining at each timepoint, with data summed from six replications of the food-leaving assay. Three replications of the experiment are not included in this figure, as they did not include all intermediate time points (all nine experiments are included in Fig 3B). Data was collected for each hour from 0–8, and at 24h. Sample size, final percent leavers, and statistical comparison are shown to the right. All subjects are males in the him-5 background. Curve comparison between genotypes performed with log-rank Mantel-Cox test. **** = p<0.0001. (TIF) [file pgen.1010273.s005.TIF]

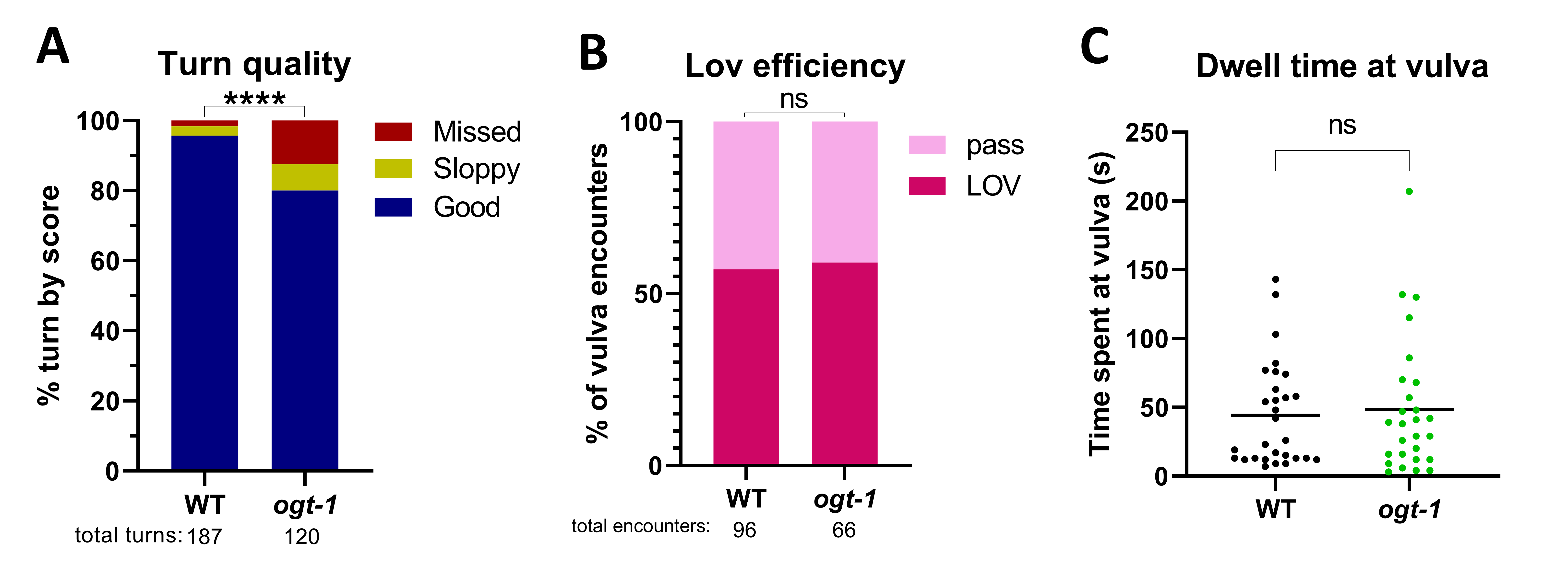

Supplement: S6 Fig — (A) ogt-1(jah01) males have a higher incidence of sloppy turns (loss of contact, but successful turn) and of missed turns (complete loss of contact with mate), as assessed by Chi-square. (B) ogt-1(jah01) males have normal vulval location efficiency, shown as the percent of vulva encounters in which the male showed successful location of vulva (LOV) versus the percent in which the male does not stop at the vulva (pass), as assessed by Fisher’s exact test. (C) ogt-1(jah01) and wild-type males dwell at the vulva for similar lengths of time, as assessed by t-test. Dwell time is defined as the time from when the male stops at the vulva to the time the male leaves the vulva. All males are in the him-5 background. **** = p<0.0001, ns = not significant. (TIF) [file pgen.1010273.s006.TIF]

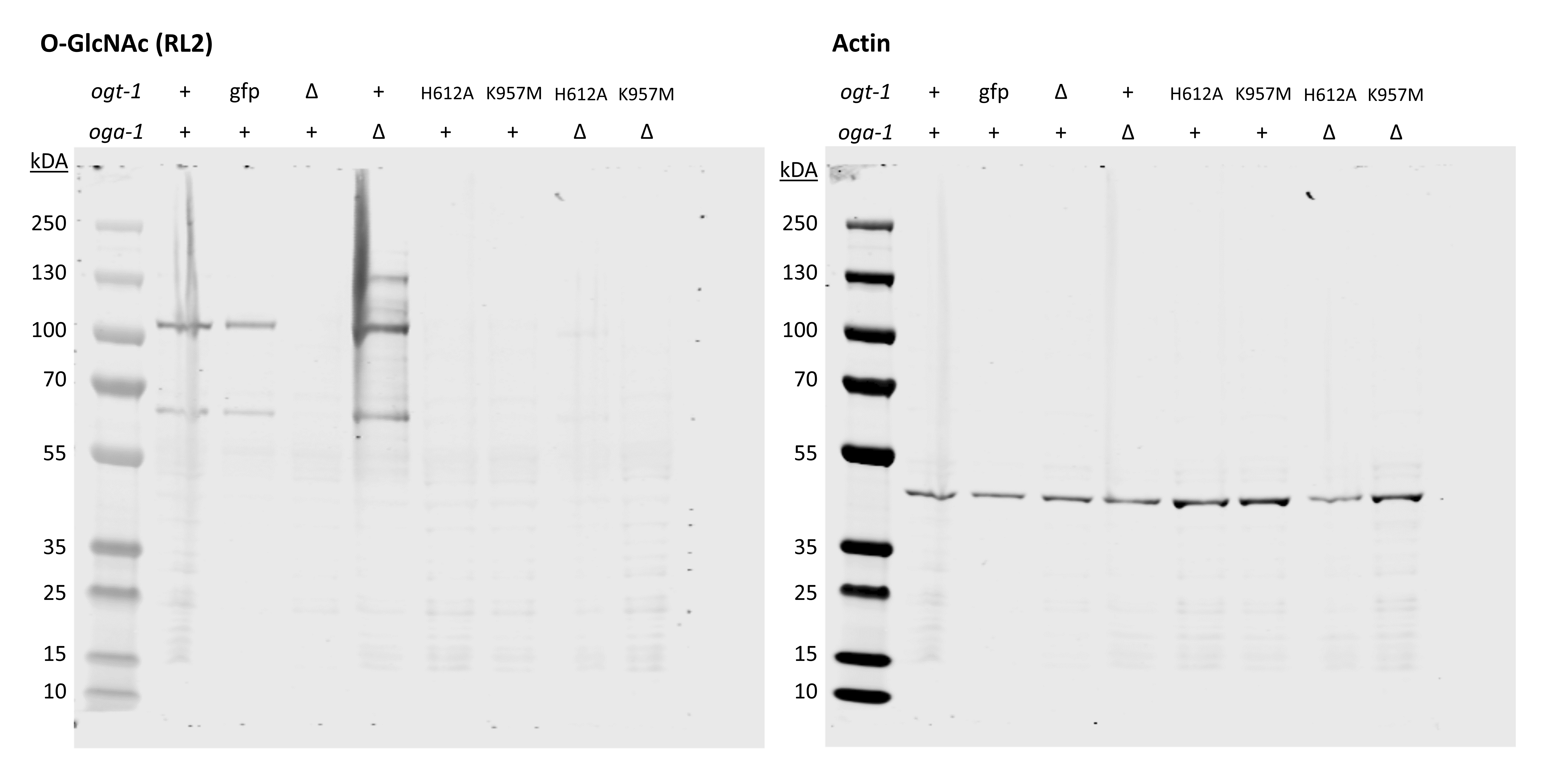

Supplement: S7 Fig — Left: O-GlcNAc (RL2 antibody), right: actin antibody. Genotypes of strains given above with regard to the ogt-1 and oga-1 genes. gfp = ogt-1(dr84), Δ = deletion (ogt-1(jah01) or oga-1(av82)), H612A = ogt-1(dr91), K957M = ogt-1(dr89). All strains are in the him-5 background. (TIF) [file pgen.1010273.s007.TIF]

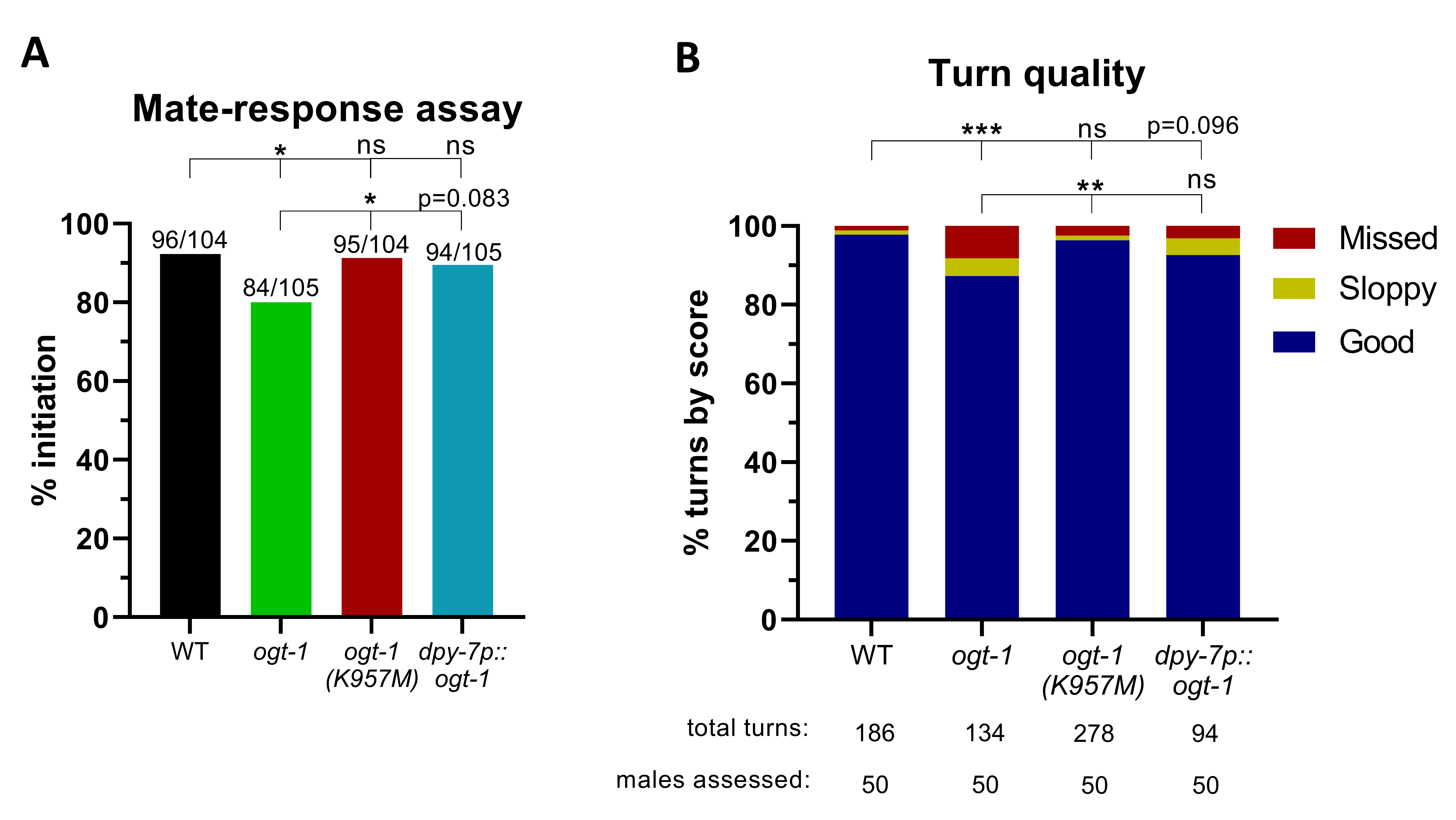

Supplement: S8 Fig — (A) Repeat of the mate-response assay, including ogt-1(K957M) and dpy-7p::ogt-1, compared with wild-type and with ogt-1(jah01) by pairwise Fisher’s exact test. Results include five independent replications of the experiment. (B) Scoring of turn quality in videos of males mating with anesthetized fem-1 animals, shown as percent of total turns assessed for each genotype. Good = successful turn with no loss of contact, sloppy = successful turn with loss of contact, missed = failed turn where male loses contact with mate. The experimenter was blinded to the genotype of the males while scoring turns. Statistical comparisons to WT and ogt-1 were performed with pairwise Chi-square analyses. * = p<0.05, ** = p<0.01, *** = p<0.001, ns = not significant. P-values between 0.05 and 0.10 are shown on the graph. ogt-1(K957M) = ogt-1(dr89). All strains are in the him-5 background, and dpy-7p::ogt-1 is additionally in the ogt-1 background. (TIF) [file pgen.1010273.s008.TIF]
