# Supplementary material for: O-GlcNAc transferase plays a non-catalytic role in C. elegans male fertility
Source: PLoS Genet. 2022 Nov 16;18(11):e1010273. doi: 10.1371/journal.pgen.1010273 (PMC9710795; doi:10.1371/journal.pgen.1010273)
Supplement: Attachment — Submitted filename: ogtfertility_ResponseToReviewers_102822.pdf [file pgen.1010273.s017.pdf]

Thank you very much for submitting your Research Article entitled 'O-GlcNAc transferase plays a non-catalytic role in C. elegans male fertility' to PLOS Genetics.
The manuscript was fully evaluated at the editorial level and by independent peer reviewers. The reviewers appreciated the attention to an important problem, but raised some substantial concerns about the current manuscript. Based on the reviews, we will not be able to accept this version of the manuscript, but we would be willing to review a much-revised version. We cannot, of course, promise publication at that time.
Should you decide to revise the manuscript for further consideration here, your revisions should address the specific points made by each reviewer. Among the points raised by the reviewers, two key issues stand out as minimal revisions that are needed for re-considering the manuscript: 1) providing more definitive results for function of ogt-1 in the hypodermis for the noted male mating phenotypes, and 2) better characterization of the mating defects (issues raised by both Reviewers #2 and #3). We will also require a detailed list of your responses to the review comments and a description of the changes you have made in the manuscript.

If you decide to revise the manuscript for further consideration at PLOS Genetics, please aim to resubmit within the next 60 days, unless it will take extra time to address the concerns of the reviewers, in which case we would appreciate an expected resubmission date by email to plosgenetics@plos.org.

If present, accompanying reviewer attachments are included with this email; please notify the journal office if any appear to be missing. They will also be available for download from the link below. You can use this link to log into the system when you are ready to submit a revised version, having first consulted our Submission Checklist.

To enhance the reproducibility of your results, we recommend that you deposit your laboratory protocols in protocols.io, where a protocol can be assigned its own identifier (DOI) such that it can be cited independently in the future. Additionally, PLOS ONE offers an option to publish peer-reviewed clinical study protocols. Read more information on sharing protocols at https://plos.org/protocols?utm_medium=editorial-email&utm_source=authorletters&utm_campaign=protocols

Please be aware that our data availability policy requires that all numerical data underlying graphs or summary statistics are included with the submission, and you will need to provide this upon resubmission if not already present. In addition, we do not permit the inclusion of phrases such as "data not shown" or "unpublished results" in manuscripts. All points should be backed up by data provided with the submission.

While revising your submission, please upload your figure files to the Preflight Analysis and Conversion Engine (PACE) digital diagnostic tool. PACE helps ensure that figures meet PLOS requirements. To use PACE, you must first register as a user. Then, login and navigate to the UPLOAD tab, where you will find detailed instructions on how to use the tool. If you encounter any issues or have any questions when using PACE, please email us at figures@plos.org.

PLOS has incorporated Similarity Check, powered by iThenticate, into its journal-wide submission system in order to screen submitted content for originality before publication. Each PLOS journal undertakes screening on a proportion of submitted articles. You will be contacted if needed following the screening process.

To resubmit, use the link below and 'Revise Submission' in the 'Submissions Needing Revision' folder.

****

We are sorry that we cannot be more positive about your manuscript at this stage. Please do not hesitate to contact us if you have any concerns or questions.

Yours sincerely,

Kaveh Ashrafi
Associate Editor
PLOS Genetics

Gregory P. Copenhaver
Editor-in-Chief
PLOS Genetics

We thank the editor for the suggestions and for the opportunity to improve our submission. We are now submitting an extensively-revised manuscript which includes several new experiments and additional data added to existing figures. Briefly, our additions include:

- Additional rescue experiment:
    - Because *dpy-7* drives expression beyond the hypodermis in the P cells, we performed a crucial control experiment testing expression in the P cells using the *hlh-3* promoter
    - Two independent *hlh-3p::ogt-1* lines do not rescue fertility, supporting the conclusion that *ogt-1* is required in the hypodermis for male fertility
- Further investigation of behavior with hypodermal rescue and catalytic-dead strains:
    - Mate-response behavior is rescuable by hypodermal *ogt-1* expression
    - Catalytic-dead males had normal mate-response and turning behavior
- Additional data and analysis of sperm:
    - Tripling the sample size for sperm count revealed a significant difference in sperm count
    - Quantification of signal from the sperm transfer assay to better illustrate this aspect of the phenotype
- Improvement of the results section to more clearly state results
- Substantial editing of the discussion section to be more succinct, clear, and to better propose hypotheses

Our detailed responses to each reviewer and comment are provided below:

Reviewer's Responses to Questions

Comments to the Authors:

Please note here if the review is uploaded as an attachment.

Reviewer #1: The manuscript “O-GlcNAc transferase plays a non-catalytic role in C. elegans male fertility” describes the reproductive defects of C. elegans males that lack the gene ogt-1, which encodes the enzyme O-GlcNAc transferase. From behavioral analyses of progeny counts, dwell times of solitary males in bacterial lawns, and visual inspection of copulation behavior, the authors found that ogt-1 deletion males have a reduced behavioral drive to initiate and sustain some of the earlier sensory-motor steps of mating. The male mating deficiencies can be partially ameliorated by copulations with mobility impaired mates, but with mobile females/hermaphrodites, the behavioral defects render many ogt-1 mutant males near infertile. The authors found that the site-of-action for OGT-1 is the male hypodermis/epidermis and interestingly, the utility of the molecule for mating behavior does not rely on its catalytic ability to transfer acetylglucosamine to other molecules. The behavioral assays are conducted and analyzed competently, and the finding that O-GlcNAc transferase-deficient OGT-1 protein can still promote male mating behavior is unexpected, exciting and implies that the molecule has additional metabolic/regulatory functions that can be further determined by studying male reproductive behavior.
I have a few very small issues that can be address via writing.

We thank the reviewer for the positive comments and constructive criticism. We have made the suggested changes to the manuscript and share data we believe will be of interest:

- Behavioral assays testing catalytic-dead mutants for mate-response and turning behavior
- Data from single male, single female mating experiments
- Fluorescence quantification of images from the sperm transfer assay

See our detailed responses to each comment below:

Page 34. Line 939. “… is placed in a small dot of OP50 (2ul) in the center…” Best describe in parentheses the small dot as a diameter of the lawn rather than a volume of inoculum.

This line (886) has been changed to: “… is placed in a small dot of OP50 (4mm diameter) in the center…”

For Figure 2 C, Need to clarify if the sperm transfer efficiency assay conducted similarly as the progeny count assay? Was this still the same 15 males to 5 female ratio for 18 hours?

We have clarified our description of this experiment in both the results and methods sections:

Line 189: “Sperm transfer can be directly assessed by tracking fluorescently labelled male-derived sperm within the reproductive tract of non-labelled mates. Unanesthetized *fem-1* worms were used as mates for consistency with the other experiments and to reduce the likelihood of ovulation which could displace male sperm. After one hour of mating, 78% of *fem-1* worms carried labelled sperm from wild-type males in their spermathecae, while only 22% of those mated with *ogt-1* males did (Fig 2C).”

Line 486: “As has been previously described [82], MitoTracker was used to stain live males and thus fluorescently label their sperm. Males were stained with 10μM

MitoTracker Red (Invitrogen) in a watch glass for two hours, then put on a normal NGM plate to recover overnight. Twenty-four males were then plated with six unlabeled mates for one hour."

Page 11 line 246. The statement " These findings suggest the later steps proceed normally in ogt-1 males, supporting…" might need to be soften a bit. The only way to make that statement confidently is to do single male/single female mating analysis in order to determine that any exceptional ogt-1(null) male that successfully intromit its spicules and transfer sperm sires a statically comparable brood relative to a single wild-type male. Most of the assays in the paper are done with couplings of 3:1 ratio of males to mates for 18 hours, so it is difficult to determine how many repeated matings (i.e with accumulated sperm) each female engaged in.

We agree with the reviewer regarding this statement, and we have cut it from the manuscript. We have tested *ogt-1* males in a 1:1 mating assay and found that while we still see a significant difference from wild-type, the majority of values for both genotypes were zero.

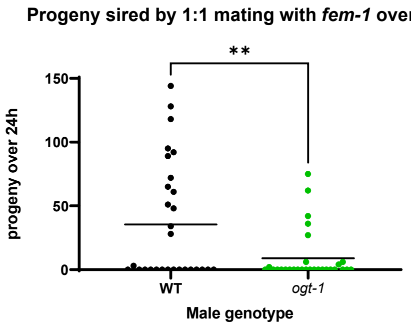

We have also analyzed the fluorescence intensity of images of *fem-1* animals following successful sperm transfers over one hour of mating with WT or *ogt-1*. This analysis revealed a significant difference in the amount of sperm transferred. We agree with the reviewer that this finding is consistent with multiple mating events occurring more frequently with WT males. We also agree that we cannot rule out the hypothesis that *ogt-1* males have a defect in insemination. This analysis has been added to S4 Fig.

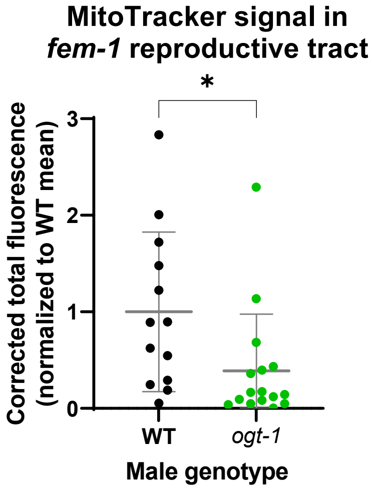

In the results or discussion, one needs to tell the reader what additional mutant phenotypes (altered lipids, altered glycogen, osmotic response differences… etc.) if any that ogt-1 catalytic dead mutants share with the ogt-1 deletion, other than the loss of sugar modification. Is mating behavior the only thing not disrupted by the catalytic point mutations? This would be important since it would allow the reader to correlate any secondary phenotypes that might impinge with copulation behavior.

So far, few *ogt-1* related phenotypes have been tested to see if catalytic activity is required. Prior to this study, only three *C. elegans* phenotypes had been tested, and all three of those did not require OGT-1 catalysis. Thus, other than the presence or absence of O-GlcNAcylation, no separation between wild-type and catalytic-dead mutants has been described. In the updated manuscript, we clearly state prior findings. The updated section from the discussion, line 358:

> "In fact, prior studies have described non-catalytic roles of OGT including aldicarb sensitivity [54], adaptation to hypertonic stress [24], and entry into adult reproductive diapause [30]. Here we have shown that OGT-1 catalysis is dispensable for normal male fertility and mating behaviors including food-leaving, mate-response, and turning (Fig 4, S8 Fig)."

In addition, we have conducted additional experiments in which we tested the catalytic-dead *ogt-1(K957M)* line for its effect on mate-response and turning behaviors, shown below. Similar to its lack of effect on progeny count, point mutation males do not differ from wild-type in either of these behaviors, as we show in S8 Fig, and discuss in the results section, line 265:

> "To further explore the behavioral phenotypes of these lines, they were tested with the mate-response assay, which showed *ogt-1* had the lowest percentage of males which responded to mates, while wild-type, *ogt-1(K957M)*, and *dpy-7p::ogt-1* had comparable proportions of worms which responded (S8A Fig). Assessment of turn quality again demonstrated *ogt-1* males had the greatest proportion of missed and sloppy turns (S8B Fig). Unlike food-leaving and mate-response, the turning behavior results for *dpy-7p::ogt-1* were closer to *ogt-1* than wild-type (S8B Fig), suggesting this behavioral defect isn't rescued by hypodermal expression of *ogt-1*. Wild-type and *ogt-1(K957M)* were roughly equivalent with very low proportions of missed or sloppy turns

(S8B Fig), demonstrating a non-catalytic mechanism is at play in each of these behaviors, as was the case with the overall progeny count (Fig 4C)."

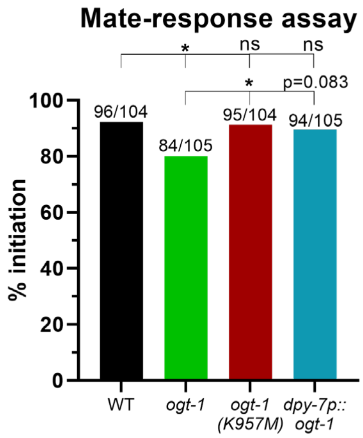

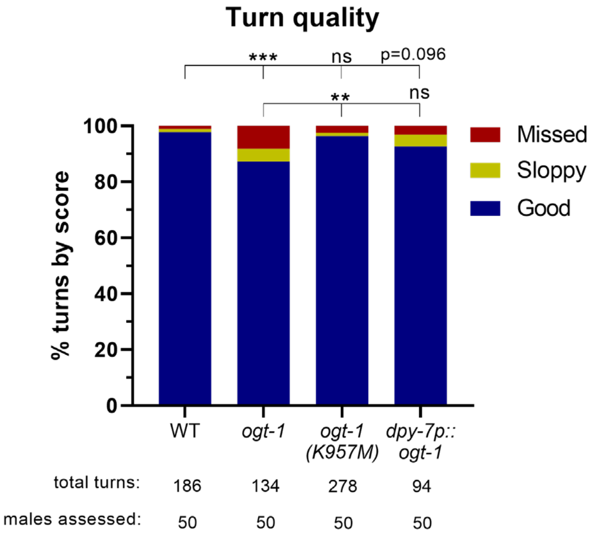

Page 19. Line 430. The statement "…suggesting the hypodermis plays a more important role in fertility than has been previously appreciated." needs to be modified. Work has been published studying how nutrition and metabolism in the hypodermis/epidermis affects C. elegans male neural muscular circuitry involved with reproductive behavior and fertility. For example, similar to the ogt-1(null) mutants, day 1 fat-6(null), fat-7(null), (delta 9 fatty acid desaturase) mutant males show higher food preference to mating than wild type males (Figure 3 using a food/mate choice assay rather than the leaving assay, in Goncalves et al. 2022 iScience 25, 104082). Like OGT-1, the fatty acid desaturase site of action is the hypodermis/epidermis.

Additionally, phosphoenolpyruvate carboxykinase, a metabolic enzyme involved in glycerol/gluconeogenesis, required for lipid and sugar stores, has a site of action that is also in the male hypodermis/epidermis, but to sustain male copulation motor responses on day 2 of adulthood, rather than on day 1 (Goncalves et al 2020. iScience 23, 100990).

We thank the reviewer for pointing out these relevant references. In the updated discussion section, we now discuss these results considering our findings.

Line 299: "The metabolic functions of the hypodermis have previously been shown to play key roles in male mating by storing and mobilizing energy to fuel neurons and muscles directly involved in mating [60, 61]."

Line 327: "Similar to the *ogt-1* food-leaving phenotype, males starved overnight were less likely to leave food in the food-leaving assay [3, 64], and the lipid-depleted *fat-6(lf);fat-7(lf)* males exhibited a behavioral preference for feeding over mating [60]. Unlike our phenotype, starving wild-type males and young adult *fat-6(lf);fat-7(lf)* males were fertile [60, 65], suggesting the *ogt-1* fertility phenotype is more complex than starvation alone."

Signed L. Rene Garcia.

Reviewer #2: This work analyses the role of ogt-1, a O-GlcNAcyl transferase, in C. elegans male fertility. The experiments show that ogt-1 mutant males shire less progeny than wildtype ones and that this is due to behavioural defects rather than sperm quality. ogt-1 males display several mating defects associated with reduced mating drive (i.e. poor response to contact and low food-leaving to explore in search of mates). Through rescue experiments, they show that ogt-1 likely acts in the hypodermis. An interesting aspect of the work, in addition, is that the reduced fertility and food leaving behaviour are independent of enzymatic activity, suggesting other important roles for this protein beyond O-GlcNAcylation. These experiments are convincingly performed through CRISPR- generated mutants and biochemical analysis of their acetylation profiles. Overall, the manuscript is rigorous, the experiments are carefully performed and the conclusions are backed up by the data. There are only a couple of points that need revisiting.

We thank the reviewer for the positive comments, and have performed additional experiments to address the suggestions, which we believe have strengthened our submission. Our new data includes:

- An additional rescue experiment addressing concerns about *dpy-7* promoter driving expression beyond the hypodermis
- Behavioral assays testing catalytic-dead mutants and hypodermal-rescue males for mate-response and turning behaviors

Please see our detailed responses to each comment below:

- The tissue-specific rescues are performed with constructs that include ogt-1 introns that are potential regulatory regions of expression. Therefore, it is unclear whether the construct driving expression under the hypodermal promoter drives expression exclusively in hypodermis or also in other tissues (intron-driven expression). So, while the conclusion that the hypodermis is necessary, is correct, it is unclear whether this is sufficient. Could the authors try rescuing with a cDNA lacking all introns so to get rid of potential regulatory regions other than the hypodermis promoter?

During our study, we created and tested *ogt-1* expression plasmids with six different promoters, of which only two were capable of rescuing fertility. We anticipate that if an element within the included introns were driving non-specific expression of *ogt-1* at levels sufficient to rescue the fertility phenotype, we would have observed higher progeny count for many lines containing these plasmids, contrary to our data.

We elected to make *ogt-1* constructs containing introns based on research which demonstrated a higher rate of expression from constructs which include one or more introns compared to those without introns (Crane et al. 2019). The endogenous introns we included (introns 5 and 6) were selected in part for their small size (50 and 52 base pairs, respectively) and their distance from the promoter, which is considered less likely to contain regulatory elements.

We agree with the reviewer that expression by the *dpy-7* promoter outside the hypodermis complicates interpretation of our results. We have now addressed this concern by testing an additional promoter (*hlh-3*) which expresses in the P cells, in which *dpy-7* also drives expression. This important control experiment shows expression in the embryonic and post-embryonic P cells is insufficient to rescue fertility. This supports the conclusion that the hypodermis is critical to this phenotype rather than other P cell descendent cells which include male tail neurons. These results are now included in the main text as Fig 1E.

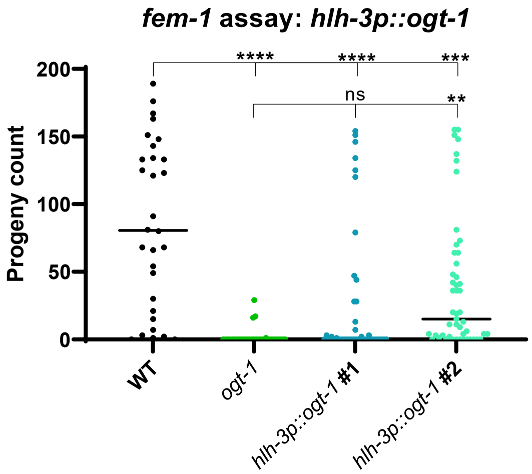

- The catalytic dead mutants should be tested for the other phenotypes identified in the ogt-1 deletion mutants (i.e. response and turns) to better dissect the contribution of the different protein domains to the whole male phenotype.

We have now tested the catalytic-dead line for its effect on mate-response and turning behavior, which is shown below and is now included in the paper as S8 Fig. Similar to its lack of effect on progeny count, point mutation males do not differ from wild-type in either of these behaviors, which we discuss in the results section, line 265:

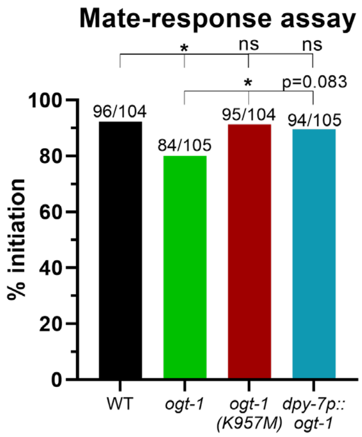

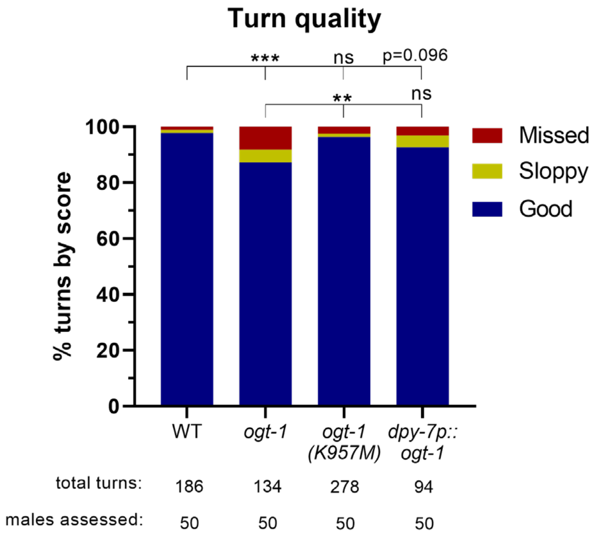

"To further explore the behavioral phenotypes of these lines, they were tested with the mate-response assay, which showed *ogt-1* had the lowest percentage of males which responded to mates, while wild-type, *ogt-1(K957M)*, and *dpy-7p::ogt-1* had comparable proportions of worms which responded (S8A Fig). Assessment of turn quality again demonstrated *ogt-1* males had the greatest proportion of missed and sloppy turns (S8B Fig). Unlike food-leaving and mate-response, the turning behavior results for *dpy-7p::ogt-1* were closer to *ogt-1* than wild-type (S8B Fig), suggesting this behavioral defect isn't rescued by hypodermal expression of *ogt-1*. Wild-type and *ogt-1(K957M)* were roughly equivalent with very low proportions of missed or sloppy turns (S8B Fig),

demonstrating a non-catalytic mechanism is at play in each of these behaviors, as was the case with the overall progeny count (Fig 4C).

- The authors show that ogt-1 males display a binomial distribution in their outcrossing success and this is due to differences in their ability to mate. Is the normalisation of response initiations per male counting all males an appropriate measure to perform and do statistical analysis, then? Since there are two different populations of males.

We share the reviewer's concern about this, and have decided to deemphasize this data in the manuscript and to move it to the supplement. The results section has been made more succinct and straightforward in its description of the behavioral results.

Reviewer #3: Konzman et al. report on the effects of the ogt-1 mutation on the reproductive performance in C. elegans males. The topic of this study is compelling because O-GlcNAc transferase is a nutrient sensor. However, I have concerns about interpretations of several experiments as well as the overall advance presented in this manuscript.

To start, dramatically reduced broods sired by ogt-1 males suggest that they have one or more substantial reproductive defects. For comparison, ogt-1 hermaphrodites suffer ~25-30% reduction in brood size, so the effect of the mutation appears to be considerably stronger in the males.

We have updated the results section of the manuscript to call attention to the difference in fertility between hermaphrodite and male *ogt-1* mutants, line 107:

> "Previous studies have shown reduced brood sizes from *ogt-1* deletion hermaphrodites, with a decrease from wild-type that ranges from 10% to 30% [20, 23, 30]. Mean *ogt-1* male brood size was 65% to 80% lower than the wild-type controls (Fig 1A)."

To narrow down the site of ogt-1 action wrt reproductive performance in males, the authors expressed ogt-1 under control of several tissue-restricted promoters. Expression in the hypodermis (dpy-7) appears to substantially rescue the phenotype. Yet, it is not clear what this result tells us because: 1) no obvious hypodermal defects are evident in ogt-1 males, 2) dpy-7 is expressed beyond the hypodermis, 3) expression in other cell types, notably the neurons, may offer at least some amelioration of the ogt-1 defect, particularly considering the variability of the test (strain #1 vs. strain #2 and individual to individual carrying the same transgene). I think a fair take on Fig. 1 is that the hypodermis plays some unclear role in the ogt-1's reproductive function, while the contributions from other cell types haven't been convincingly ruled out.

We agree with the reviewer's concern that the *dpy-7* promoter expresses beyond the hypodermis and have addressed this with a new rescue experiment. We constructed two independent lines which express *ogt-1* in the embryonic and post-embryonic P cells (overlapping with early *dpy-7p::ogt-1* expression), but not in the hypodermis, and tested their fertility. The fertility results are shown below and are now included in the manuscript as Fig 1E. Also included is the updated results section where we describe these findings, line 143:

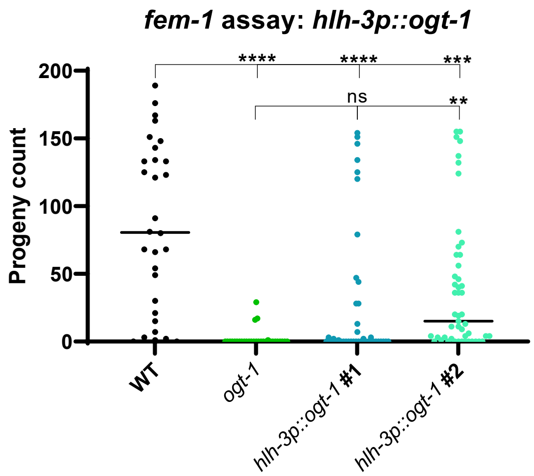

The *dpy-7* promoter is frequently used to drive hypodermal expression in tissue-specific rescue experiments [41, 44], but careful analysis of *dpy-7* expression has shown it turns on in embryonic and post-embryonic P cells [45]. Through development, the P cells produce many daughter cells which differentiate into both hypodermal cells and neurons [46]. Of particular interest are P10 and P11, which in the male produce several cells that form the hook sensillum and other male tail neurons [46]. This suggests the possibility that in the *dpy-7p::ogt-1* rescue line, early expression of *ogt-1* in P10 and P11 could be inherited by neuronal daughter cells which ultimately play a critical role in the adult male tail. We tested this idea genetically with an additional rescue experiment, selecting the *hlh-3* promoter, which expresses in the early P cells like the *dpy-7* promoter, but not in hypodermal cells later in development [47]. Two independent *hlh-3p::ogt-1* lines showed fertility significantly lower than wild-type (Fig 1E), indicating a failure of P cell expression to rescue the *ogt-1* fertility phenotype. With the finding that the pan-neuronal promoters of *rgef-1* and *unc-119* also did not rescue fertility (Fig 1C), this suggests it is unlikely that *ogt-1* contributes to male fertility through its expression in P cell descendent neurons. These data support the interpretation that the *dpy-7p::ogt-1* construct rescues fertility due to its expression in the hypodermis, not its expression in the early P cells.

We have also updated a section in the discussion to point out that while *ogt-1* mutants don't have clear morphological phenotypes such as Dpy which are associated with the hypodermis, two prior studies have demonstrated functional roles of *ogt-1* in the tissue, line 289:

Previously, *ogt-1* was shown to be required in the hypodermis for the adaptation of worms to hypertonic environmental stress [24]. A screen for genes involved in preventing transdifferentiation identified *ogt-1* as a gene that helps maintain the hypodermal cell fate [32], a role it also plays in other tissues [33], though whether this has bearing on the mutant phenotypes is unclear.

The authors next focus on the idea that behavioral defects are the cause of reduced reproductive performance of ogt-1 males. The conclusion in line 185 "initiation of mating was a significant barrier to reproduction for ogt-1 males" does not appear to be warranted by the results. Given the data presented up to this point, it is possible that the brood size defect of ogt-1 males is due to inability to sustain mating, rather than to initiate it.

This sentence has been removed.

The results described in lines 193-194 and presented in Fig. 2D do not rule out sperm defects. First, based on the description provided, we are left to assume that all germline nuclei were counted. Second, there appears to be a reasonable indication that ogt-1 males have fewer germline nuclei, and that the lack of significance is due to high interindividual variability and very low sample size. Third, even if ogt-1 males have approx. as many sperm as WT, those sperm could have substantial defects, for instance in movement or guidance.

We have analyzed additional images of DAPI-stained virgin males to triple the sample size from our initial submission. The reviewer is correct that there are fewer nuclei present in *ogt-1* male germlines which is evident with the new data, shown below and added to Fig 2D. We also agree with the reviewer that other sperm defects independent of sperm count are not ruled out by our data, and state this more clearly in the manuscript. In addition, we have quantified the MitoTracker fluorescence

in images of *fem-1* worms following successful sperm transfer from either WT or *ogt-1* males, shown below and added to S4 Fig. This analysis shows a significant difference between WT and *ogt-1* males, suggesting fewer sperm are transferred by *ogt-1* males. The relevant portions of the results section (starting line 203) and the discussion section (starting line 345) are copied below.

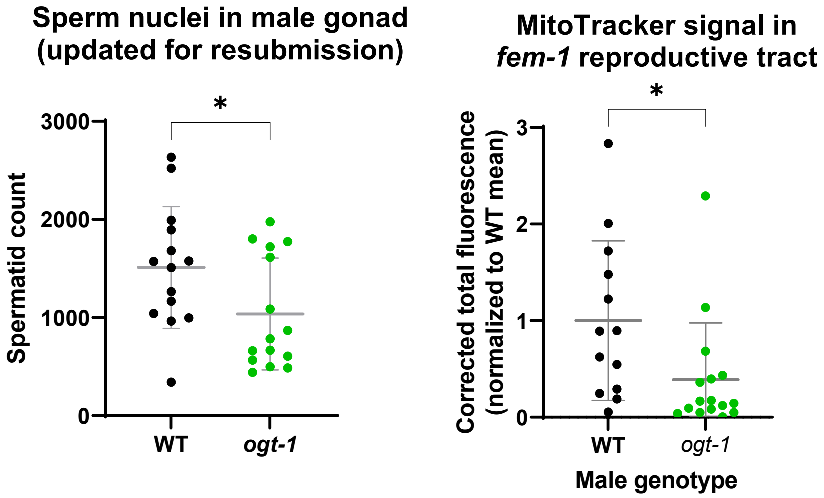

Line 194: "Images of the *fem-1* worms following successful sperm transfer showed there was typically less fluorescent signal present in worms mated with *ogt-1* males (S4 Fig), suggesting fewer sperm were transferred. Labelled sperm from either genotype appeared concentrated in the spermatheca (S4 Fig), consistent with *ogt-1* sperm correctly localizing to the spermatheca as would be expected of sperm with normal activation, guidance, and motility. Analysis of high-resolution z-stacks of DAPI stained virgin males revealed the number of spermatids in *ogt-1* males averages just over 1,000, about one third less than the wild-type average near 1,500 (Fig 2D)."

Line 335: "We found that prior to mating, young adult *ogt-1* males had a sperm count significantly lower than wild-type (Fig 2D). Still, *ogt-1* males had an average sperm count over one thousand, which suggests this is unlikely to fully explain the low progeny counts we measured for overnight mating (Fig 1A). In fact, this reduction is of similar magnitude to the reduction in hermaphrodite brood size that has been reported for the *ogt-1(ok430)* strain [23], raising the possibility that reduced sperm count is a phenotype common to males and hermaphrodites. It remains possible *ogt-1* sperm are defective in some way, as we did not directly test their activation, guidance, motility, or ability to fertilize oocytes. However, a sperm transfer assay suggested *ogt-1* male sperm are capable of properly localizing to the spermatheca (S4 Fig)."

The paragraph that starts on line 207 seemingly interchangeably uses terms "leaving assay", "searching behavior", and "exploratory behavior". Since the equivalence between these terms has not been firmly established, it would be best to refer to the results of the assay as such.

The inference (line 215) that the somewhat higher % of leavers among ogt-1 males is due to "reduced mating drive" is not supported by the data presented in the preceding section, because this experiment did not test mating drive.

The inference (line 222) that the observation of fewer initiated matings by ogt-1 males is due to the fact that "ogt-1 males don't seek mates as actively as wild-type" conflates results of the experiment described in the paragraph that starts on line 216 with the previous experiment. This conclusion does not appear to be warranted by the data.

We have gone through our manuscript to clarify our introduction to the leaving assay and the terms we use in discussing the results. For clarity and consistency, we now always refer to the assay as the "food-leaving assay", and now only discuss our interpretation regarding sex drive in the discussion section. We have removed the terms "exploratory behavior" and "mating drive" from the manuscript.

We based our food-leaving assay and our use of these terms after the first published paper to use the leaving assay, Lipton 2004. The Lipton paper and other studies using this assay frequently refer to the results as "leaving behavior", "mate-searching behavior", and "exploratory behavior" interchangeably. See also Barrios 2014, a review paper which discusses the findings various labs have contributed using the leaving assay.

I found the separation of Table 1 vs. Fig. 4A, 4C to be somewhat confusing. The authors do explain the difference between the data presented in the figure and the table, but perhaps there is a way to streamline this part of the presentation.

We thank the reviewer for this feedback and agree this section was confusing and contributed little to the overall conclusions. We have now streamlined our presentation of these results such that this section is much shorter and more declarative. What were Fig 4 and Table 1 in the original submission are now in the supplement.

It seems that in Fig. 4A the two distributions are quite similar, but the top five values for WT shift the mean upward. Is this the case?

We agree with the reviewer's assessment that the differences in "turns per male" were minor and biased by a handful of wild-type outliers. As such, we have decided to remove this figure from the manuscript.

Were experiments in Fig. 4A and, particularly, Fig. 4B blinded?

In the initial submission, the experimenter scoring turning behavior was not blinded. We have addressed this concern by blinding the scorer to the genotype of males when we performed the additional turning experiment. Encouragingly, this new experiment showed similar results for wild-type and *ogt-1* as we described for the initial experiment, suggesting scoring was similar with or without blinding. See the new turn quality figure (S8B Fig), copied in the response to the next comment.

The manuscript ends with an intriguing suggestion that the reproductive defects in males may be due to ogt-1 functions that do not depend on the catalytic domain, but it remains unclear what those functions may be.

To better understand the effects of catalysis on the mating process, we assessed the effect of catalytic-dead *ogt-1* on response and turning behaviors, shown below. These results show no difference between *ogt-1(K957M)* and wild-type, correlating with progeny count and the food-leaving assay.

In the discussion, we discuss what is known about non-catalytic functions of OGT and suggest its role in fertility is likely due to its interactions with other proteins.

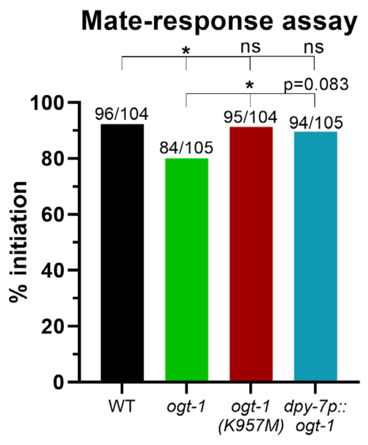

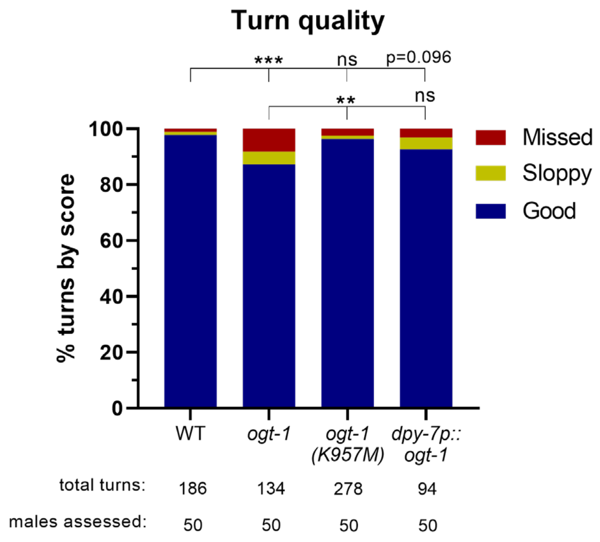

My overall impression of the manuscript is that it highlights an interesting phenotype – a largely male-specific fecundity defect in ogt-1 mutants – that is caused by largely unclear mechanism(s). It could be due to gamete defect(s) as well as several behavioral defects acting in unknown cells. Given the broad expression of ogt-1, this remains perhaps the most plausible interpretation of the results presented in this manuscript. Overall, this study offers some, but modest insights into the mechanisms by which ogt-1 acts in male reproduction in C. elegans.

We thank the reviewer for the insightful review which has helped us to greatly improve both our data and the manuscript. Our study provides several important insights:

- Loss of *ogt-1* leads to low fertility
- Hypodermal expression of *ogt-1* can rescue fertility
    - *hlh-3p::ogt-1* confirms expression outside of the hypodermis is insufficient for rescue
- *ogt-1* male sperm count is reduced
- Aberrant mating behavior may cause low fertility
    - *ogt-1* males produce more progeny count with anesthetized mates
    - WT, *ogt-1(K957M)*, and *dpy-7p::ogt-1* show similar fertility and behavior
- Catalytic activity of OGT-1 is not required for fertility

Minor comments:

Figures did not render well in the PDF version of the manuscript. This made it difficult to follow the finer points depicted in those figures.

The PDF is automatically generated from the files we submit, and upon resubmission we will do our best to ensure the generated PDF has better-looking figures. Downloading the .tiff files in our submission should provide high-quality images regardless of the quality of the PDF images.

The manuscript could benefit from additional editing. Two examples to illustrate this point - "the many different tissues" (Line 124) and "To determine which tissue ogt-1 expression is critical" (Line 124). There are several others.

We thank the reviewer for pointing out these typos. We have corrected these and others throughout the manuscript.

At seven pages, the Discussion section is as long as the Results section.
We have edited the discussion section such that it is two pages shorter than it was in our initial submission, resulting in a more focused and succinct discussion.

Part of the manuscript's motivation seems to be the use of C. elegans ogt-1 as a model for human O-GlcNAc transferase. It is not clear how well the phenotypes discussed in this manuscript advance this case. It may be better to focus on deeper understanding of the role(s) of ogt-1 in male fertility in C. elegans.
We appreciate this suggestion and have removed much of our discussion of human disease. Though we discuss findings from other systems in our discussion section, this has been pared down to focus more on *C. elegans* male fertility.
